# Affection of Peyer’s Glands in Adynamic Fever

**Published:** 1847-08

**Authors:** 


*Affection of Peyer's Glands in Adynamic Fever.*—At a meeting of the New-York Medical and Surgical Society, January 23d, Dr. Swett stated that he had lately had three cases of typhus, throwing some light upon the pathology of the disease, especially as to the condition of Peyer's glands. The patients arrived in a vessel in which the disease had been very violent, the captain and all the sailors having suffered from it. In the first patient, no ulcerations were discovered, but some patches of Peyer's glands, near the ileo-cœcal valve, were enlarged and discolored, being of reddish brown hue. In the second, a girl of 20, who had the ordinary symptoms, the glands of Peyer were very much inflamed and ulcerated. The third case was that of a boy, attacked in a week after landing, who had suffered much privation. At the time of his entrance into the hospital, he was exceedingly prostrated. Stimuli were given at once, and he improved considerably. He lived for a fortnight afterwards, and for the last week seemed constantly dying. A day or two before his death, livid discolorations of a linear form appeared about the upper part of the abdomen, as if from the bursting of small veins. On post-mortem examination, no disease of Peyer's glands, whatever, was discovered, except that some of them were hypertrophied, the results, no doubt, of former disease. In the cœcum, there were about a dozen sloughing ulcers, irregular in shape, the largest of them about the size of a five-cent piece. Taking the three cases together, it appears that the disease, although of common origin, may or may not be attended with ulceration of Peyer's glands. This is in accordance with opinion of the English, and opposed to that of the French pathologists.—*New-York Annalist.*

